# NK and CD8+ T cell phenotypes predict onset and control of CMV viremia after kidney transplant

**DOI:** 10.1172/jci.insight.153175

**Published:** 2021-11-08

**Authors:** Harry Pickering, Subha Sen, Janice Arakawa-Hoyt, Kenichi Ishiyama, Yumeng Sun, Rajesh Parmar, Richard S. Ahn, Gemalene Sunga, Megan Llamas, Alexander Hoffmann, Mario Deng, Suphamai Bunnapradist, Joanna M. Schaenman, David W. Gjertson, Maura Rossetti, Lewis L. Lanier, Elaine F. Reed, CMV Systems Immunobiology Group

**Affiliations:** 1 Department of Pathology and Laboratory Medicine, University of California, Los Angeles, Los Angeles, California, USA.; 2 Department of Microbiology and Immunology, Parker Institute for Cancer Immunotherapy, University of California, San Francisco, San Francisco, California, USA.; 3 Microbiology, Immunology, and Molecular Genetics,; 4 Institute for Quantitative and Computational Biosciences, and; 5 Division of Infectious Diseases, Department of Medicine, University of California, Los Angeles, Los Angeles, California, USA.; 6 Division of Nephrology, David Geffen School of Medicine, Los Angeles, California, USA.; 7 Biostatistics, University of California, Los Angeles, Los Angeles, California, USA.; 8 The CMV Systems Immunobiology Group is detailed in Supplemental Acknowledgments.

**Keywords:** Immunology, Transplantation, NK cells, Organ transplantation, T cells

## Abstract

CMV causes mostly asymptomatic but lifelong infection. Primary infection or reactivation in immunocompromised individuals can be life-threatening. CMV viremia often occurs in solid organ transplant recipients and associates with decreased graft survival and higher mortality. Furthering understanding of impaired immunity that allows CMV reactivation is critical to guiding antiviral therapy and examining the effect of CMV on solid organ transplant outcomes. This study characterized longitudinal immune responses to CMV in 31 kidney transplant recipients with CMV viremia and matched, nonviremic recipients. Recipients were sampled 3 and 12 months after transplant, with additional samples 1 week and 1 month after viremia. PBMCs were stained for NK and T cell markers. PBMC transcriptomes were characterized by RNA-Seq. Plasma proteins were quantified by Luminex. CD8^+^ T cell transcriptomes were characterized by single-cell RNA-Seq. Before viremia, patients had high levels of IL-15 with concurrent expansion of immature CD56^bright^ NK cells. After viremia, mature CD56^dim^ NK cells and CD28^–^CD8^+^ T cells upregulating inhibitory and NK-associated receptors were expanded. Memory NK cells and NK-like CD28^–^CD8^+^ T cells were associated with control of viremia. These findings suggest that signatures of innate activation may be prognostic for CMV reactivation after transplant, while CD8^+^ T cell functionality is critical for effective control of CMV.

## Introduction

CMV infection in immunocompetent individuals is mostly asymptomatic (1), but the virus is controlled rather than cleared, leading to lifelong persistence (2). Infection induces humoral immunity and circulating anti-CMV antibodies are used to define prior exposure (3); however, the protective role of these antibodies is unclear. Conversely, NK and T cells, particularly CD8^+^ T cells, are critical for control of primary infection and reactivation (4, 5). CMV leads to long-term changes in both these cellular populations. CMV drives expansion of NKG2C^+^ memory-like NK cells (6) and terminally differentiated CD8^+^ T cells (7, 8), the latter population further increases in abundance with each reactivation and with age, presumably due to persistent antigen exposure, in a process known as memory inflation (9, 10).

In immunocompromised individuals, reactivation of CMV is common and often leads to symptomatic infection. Similarly, immunocompromised individuals ineffectively control primary infection with more severe outcomes (11), ranging from fatigue and malaise to tissue-invasive infections, with potentially fatal outcomes. CMV primary infection or reactivation is a significant problem in transplant recipients receiving immunosuppressive therapy, with direct effects from CMV disease and indirect immunopathological effects (12). In solid organ transplant, CMV viremia and symptomatic disease are associated with chronic graft dysfunction and rejection (12), and NK and CD8^+^ T cell number and activity have been implicated in control of CMV after transplant and graft outcome (13–16). For this reason, transplant recipients receive antiviral prophylaxis with additional routine screening for CMV DNA in the periphery to monitor control of infection (17).

**Figure 1:**
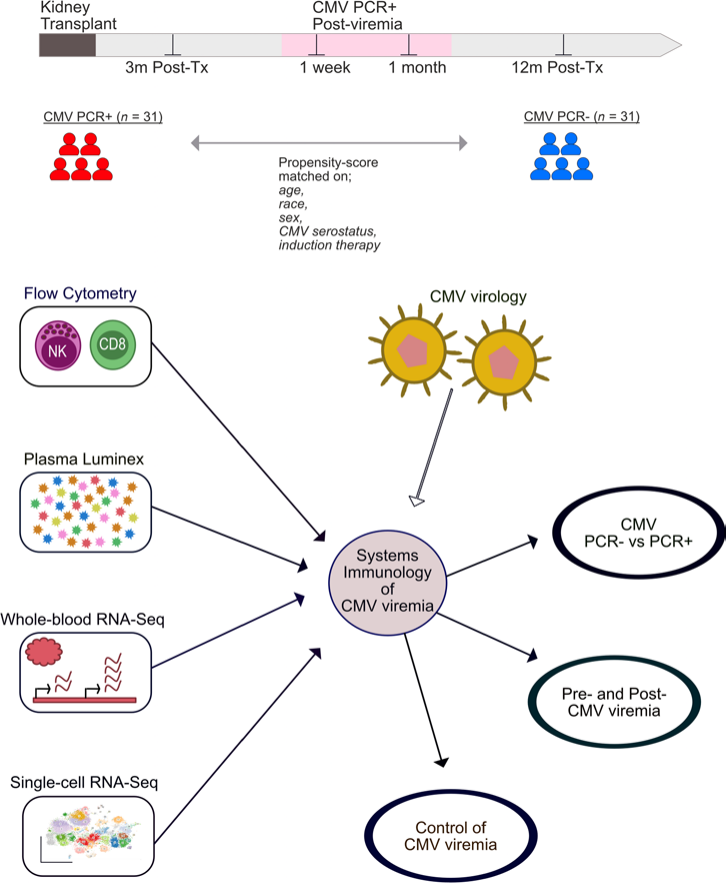
Schematic of study design, systems immunology approach, and integrated, multiomic analysis.

Despite improvements in antiviral prophylaxis and routine clinical practices, CMV viremia remains a major problem in solid organ transplant, and the host factors that allow primary infection and reactivation are incompletely understood. This study enrolled kidney transplant recipients prior to viremia, acutely after viremia, and long-term post-CMV viremia and propensity-matched nonviremic controls. Utilizing a systems immunological approach, we undertook longitudinal profiling of circulating NK and T cells, plasma chemokines and cytokines, and whole-blood transcriptional modules to further our understanding of factors predictive of onset and/or control of CMV viremia in vulnerable patients following kidney transplantation. We additionally generated longitudinal single-cell transcriptomes of CD8^+^ T cells to provide higher resolution information on these critically important cells in control of CMV (Figure 1).

## Results

### CMV PCR^+^ and CMV PCR^–^ cohort clinical characteristics.

The demographic and clinical variables of the study population are summarized in Table 1. The proportion of living donors and frequency of rejection and graft status at 1 year after transplant were equivalent between CMV PCR^+^ and PCR^–^ patients. CMV PCR^+^ patients were sampled approximately 3 months after transplant and before viremia, 1 week (median, 11 days after detection of viremia) and 1 month (median, 52 days after detection of viremia) after viremia, and 12 months after transplant (median, 205 days after detection of viremia). For PCR^+^ patients the sample before viremia was obtained approximately 1 month prior to detection of CMV viremia (median, 34.5 days). CMV PCR^–^ patients were sampled 3 and 12 months after transplant. Circulating cellular and soluble immunophenotypes of each patient were profiled longitudinally to identify integrated signatures of different stages of CMV viremia and, ultimately, effective control of the virus (Figure 1).

### Frequency of CD56^bright^ NK cells associates with detection and control of CMV viremia.

**Figure 2:**
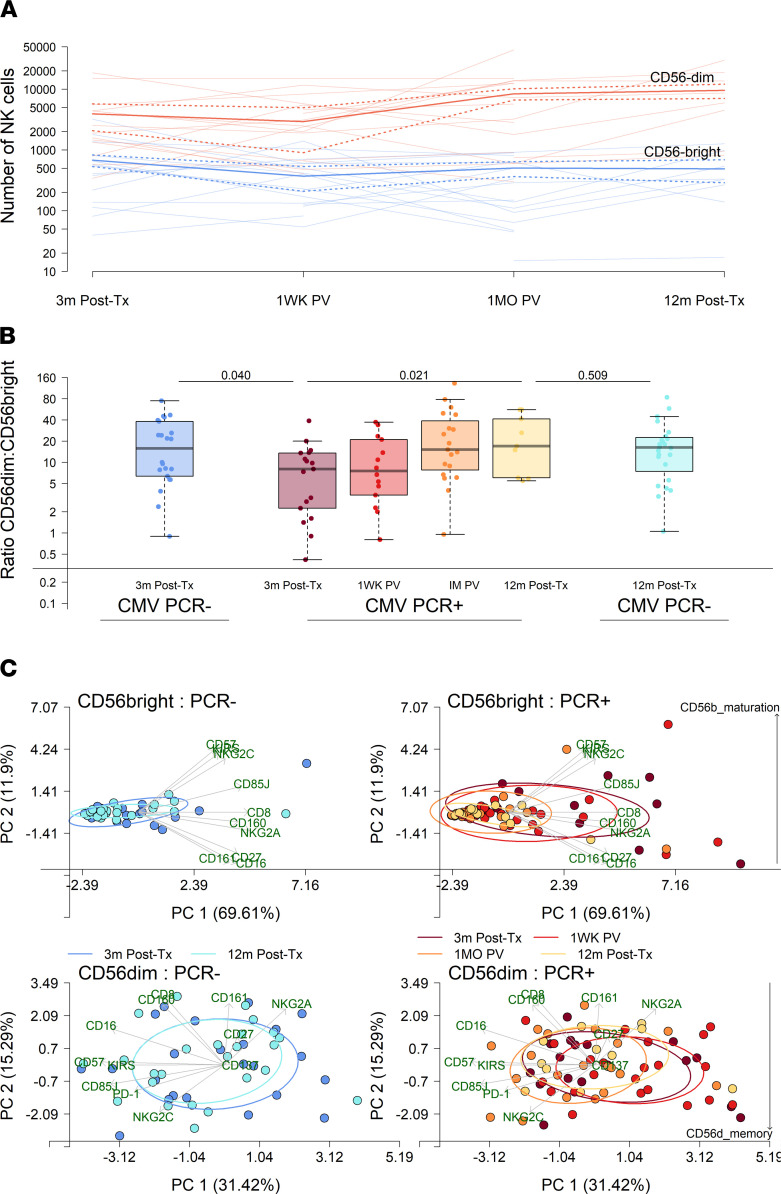
CD56 expression on NK cells delineates stages of CMV viremia. (**A**) Median number of CD56^bright^ (blue) and CD56^dim^ (red) NK cells at 3 months after transplant (post-Tx), before viremia, and longitudinally after detection of viremia; 2 standard deviations (dotted lines) and numbers per patient (nonbold lines) are indicated. (**B**) Ratio of CD56^dim^/CD56^bright^ NK cells in CMV PCR^–^ patients 3 months (dark blue, *n* = 17) and 12 months after transplant (light blue, *n* = 24) and CMV PCR^+^ patients 3 months after transplant (purple, *n* = 14), 1 week after viremia (1WK PV, red, *n* = 19), 1 month after viremia (1M PV, orange, *n* = 9), and 12 months after transplant (yellow, *n* = 22). *P* values comparing CMV PCR^–^ and CMV PCR^+^ at 3 and 12 months after transplant, determined by binomial logistic regression, and change over time after detection of viremia in CMV PCR^+^ patients, determined by linear regression, including patient ID as a random effect, are shown. (**C**) Principal component analysis of individual marker expression values on CD56^bright^ (blue) and CD56^dim^ (red) NK cells. Ellipses represent 50% of patient variance per group, with increasing width of lines indicating increasing time after transplant for CMV PCR^–^ patients and time after transplant and after detection of viremia for CMV PCR^+^ patients. Direction and strength of variance explained by each marker is indicated by annotated green arrows. Increasing evidence of CD56^bright^ NK cell maturation (CD56b_maturation) and CD56^dim^ NK cell memory-like (CD56d_memory) phenotypes are highlighted.

Immunophenotyping of peripheral blood showed that the majority of circulating NK cells are mature CD56^dim^ cells. The absolute number of CD56^dim^ NK cells was increased 1 month after detection of CMV viremia and 12 months after transplant in PCR^+^ patients; conversely, immature CD56^bright^ NK cells decreased 1 week after detection of CMV viremia (Figure 2A). Similarly, the ratio of CD56^dim^ to CD56^bright^ NK cells increased significantly after viremia (Figure 2B). This was still true after adjusting for days after transplant at which CMV viremia was first detected (*P* = 0.013) and whether the recipient had a confirmed rejection (*P* = 0.018). The ratio was significantly lower 3 months after transplant before the detection of viremia in PCR^+^ patients compared with PCR^–^ controls, whereas the ratio was equivalent 12 months after transplant. Principal component analysis of the surface marker profile of these cells further highlighted a contraction of immature CD56^bright^ NK cells after viremia, illustrated by contraction of ellipses over time, with continued low expression of markers of maturation (CD56b_maturation), such as CD57 and killer cell immunoglobulin-like receptors (KIRs) (Figure 2C). The phenotype of CD56^dim^ NK cells was less affected by CMV viremia, although there was a nonsignificant decrease in NK cells with markers of memory (CD56d_memory), most notably NKG2C. When CMV-seronegative and -seropositive recipients were analyzed separately, we consistently found a reduced ratio of CD56^dim^ to CD56^bright^ NK cells at 3 months after transplant in PCR^+^ individuals (Supplemental Figure 1; supplemental material available online with this article; https://doi.org/10.1172/jci.insight.153175DS1).

### CMV viremia drives expansion of terminally differentiated CD8^+^ T cells.

**Figure 3:**
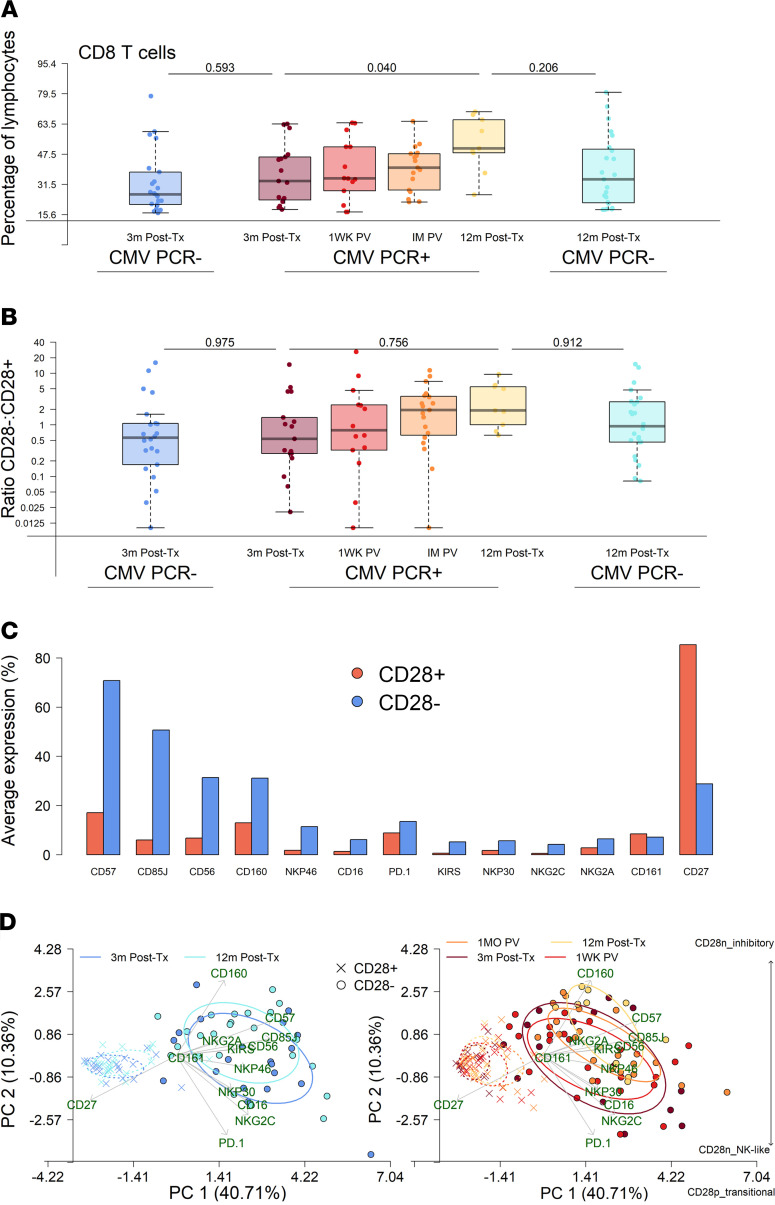
CD8^+^ T cells expressing inhibitory and NK-like receptors increase after CMV viremia. (**A**) CD8^+^ T cells as a percentage of lymphocytes for CMV PCR^–^ patients 3 months (dark blue, *n* = 17) and 12 months after transplant (light blue, *n* = 24) and CMV PCR^+^ patients 3 months after transplant (purple, *n* = 14), 1 week after viremia (1WK PV, red, *n* = 19), 1 month after viremia (1M PV, orange, *n* = 9),and 12 months after transplant (yellow, *n* = 22). *P* values comparing CMV PCR^–^ and CMV PCR^+^ at 3 and 12 months after transplant, determined by binomial logistic regression, and change over time after detection of viremia in CMV PCR^+^ patients, determined by linear regression, including patient ID as a random effect, are shown. (**B**) Ratio of CD28^–^/CD28^+^ CD8^+^ T cells as described above. (**C**) Mean percentage of CD28^–^ (blue) and CD28^+^ (red) T cells expressing each marker. (**D**) Principal component analysis of individual marker expression values on CD28^–^ (blue) and CD28^+^ (red) T cells. Ellipses represent 50% of patient variance per group, with increasing width of lines indicating increasing time after transplant for CMV PCR^–^ patients and time after transplant and after detection of viremia for CMV PCR^+^ patients; full and dashed lines represent CD28^–^ and CD28^+^ cells, respectively. Direction and strength of variance explained by each marker is indicated by annotated green arrows. Increasing evidence of transitional (CD28p_transitional), inhibitory (CD28n_inhibitory), and NK-like (CD28n_NK-like) phenotypes are highlighted.

CD8^+^ T cell abundance, as a proportion of peripheral lymphocytes, was equivalent 3 months after transplant between CMV PCR^+^ and PCR^–^ controls (Figure 3A). CD8^+^ T cells were significantly expanded by CMV viremia, with an increased proportion 1 month after viremia and 12 months after transplant. This was still true after adjusting for days after transplant at which CMV viremia was first detected (*P* = 0.033) and whether the recipient had a confirmed rejection (*P* = 0.032). To differentiate early and later stages of differentiation (18), CD8^+^ T cells were defined as either CD28^+^ or CD28^–^. Similar to total CD8^+^ T cells, the ratio of CD28^–^ to CD28^+^ cells was equivalent 3 months after transplant but increased after viremia and was higher still at 12 months after transplant (Figure 3B). This ratio increased from 3 to 12 months after transplant in CMV PCR^–^ controls, but the magnitude of change was not as pronounced as for those who experienced CMV viremia. Twelve months after transplant the median percentage of CD28^–^CD8^+^ T cells was 65.5% for CMV PCR^+^ patients (IQR, 50.0%–84.6%), but only 48.2% for CMV PCR^–^ patients (IQR, 31.7%–73.5%). As expected, the percentage of CD28^–^CD8^+^ T cells was increased with age at 3 months after transplant in CMV PCR^–^ and PCR^+^ patients; however, the described after viremia increase was not associated with age (Supplemental Figure 2). An early differentiation phenotype of CD28^+^ cells was confirmed by a high percentage of cells expressing CD27 (Figure 3C). Conversely, CD28^–^ cells were defined by low expression of CD27, with a high percentage of cells expressing terminal differentiation and inhibitory receptors, including CD57 and CD85j, and NK-associated receptors, including CD56 and NKp46. These findings were supported by principal component analysis of expression of all surface markers together (Figure 3D). The phenotype of CD28^–^ and CD28^+^ cells did not change from 3 to 12 months after transplant in CMV PCR^–^ patients. In contrast, the phenotype of CD28^–^ T cells was dramatically altered after viremia, most notably upregulating CD57 and CD160, illustrated by increasing PC2 scores of ellipses over time after viremia. When CMV-seronegative and -seropositive recipients were analyzed separately, we consistently found an increasing ratio of CD28^–^ to CD28^+^CD8^+^ T cells after viremia in PCR^+^ individuals (Supplemental Figure 3).

### Expanded CD28^–^CD8^+^ T cells express markers of cytotoxicity and NK-like phenotype.

**Figure 4:**
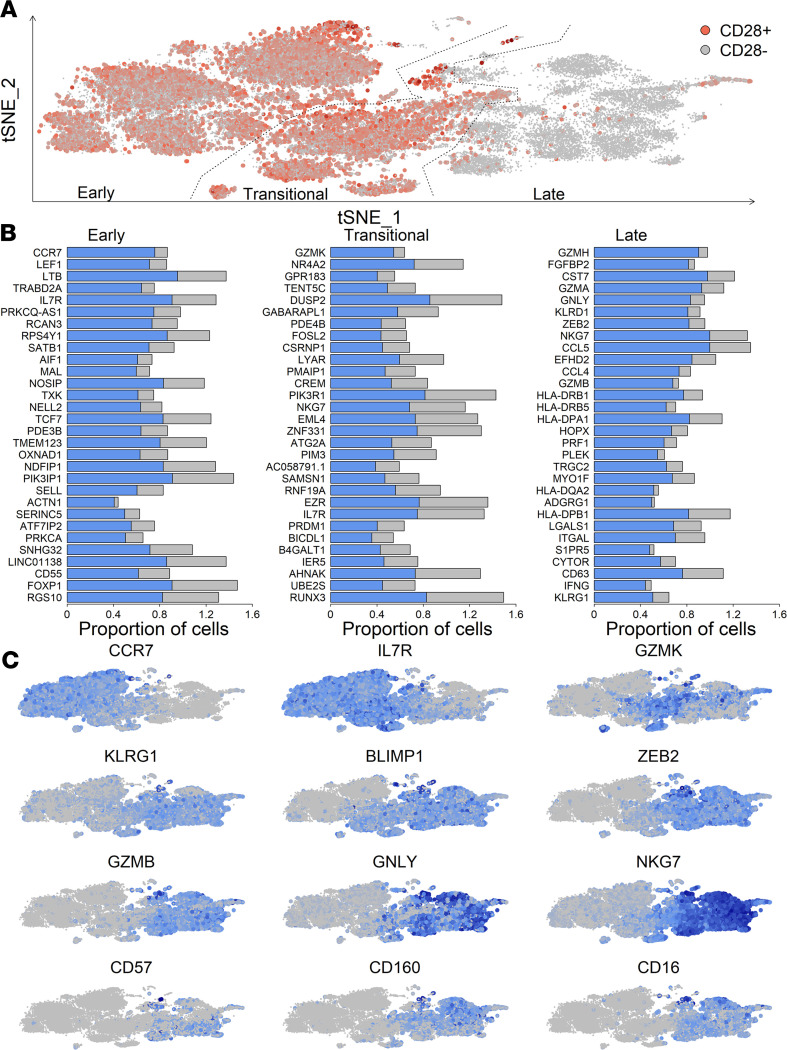
Cytotoxic, inhibitory, and NK-like phenotype of CD28^–^CD8^+^ T cells highlighted by single-cell transcriptomics. (**A**) Single-cell transcriptomes of CD8^+^ T cells, purified by negative selection, were reduced to 2 dimensions by t-SNE for visualization. Cells were classified as early, transitional, or late by expression of CD28 and known markers of CD8^+^ differentiation. (**B**) Differentially expressed transcripts per classification. Proportion of cells expressing each transcript within each class (blue) and proportion expressing each transcript outside each class (gray) are shown. (**C**) Normalized expression of transcripts delineating early, transitional, and late differentiated CD8^+^ T cells, from low (gray) to high (dark blue).

Given the observed changes in frequency and phenotype of CD8^+^ T cells, we performed single-cell transcriptomics on purified CD8^+^ T cells from a subset of 6 patients to comprehensively profile their expression profile. Single-cell transcriptomes from 34,182 CD8^+^ T cells were used to identify 43 clusters of CD8^+^ T cells, and complete expression profiles were reduced to 2 dimensions by t-SNE for visualization of cells. Focusing on expression of *CD28*, along with other canonical markers of CD8^+^ T cell differentiation, cells were defined as being at early, transitional, or late stages of differentiation (Figure 4A). Early-stage cells were CD28^+^ and included 17 clusters, with the majority of cells expressing markers of naive state (19), including *CCR7*, *IL7R* (CD127), and *TCF7* (Figure 4B). Late-stage cells were CD28^–^ and included 13 clusters, with downregulation of aforementioned naive-associated markers and upregulation of cytotoxicity, inhibitory, and NK-associated transcripts, suggesting an advanced differentiated phenotype. Transitional-stage cells were CD28^+^ and included 13 clusters; they shared characteristics of both early- and late-stage cells. Coexpression of *PRDM1* (BLIMP-1), *KLRG1,* and *GZMK* (granzyme K) suggested that a large proportion of transitional cells were likely short-lived effector cells (20–22). Overlaying transcript expression on all cells highlighted *GZMB* (granzyme B), *GNLY* (granulysin), and *NKG7* as being expressed on all late-stage cells, whereas *CD57*, *CD160,* and *CD16* were expressed on subsets of these cells (Figure 4C).

T cell exhaustion is commonly identified in chronic infections. To examine for evidence of exhausted cells in our study cohort, we identified cells coexpressing ≥3 of the following exhaustion-associated genes: *HAVCR2* (TIM-3), *LAG3, PDCD1* (PD-1), and *TOX*. Around 1% of total CD8^+^ cells had a putatively exhausted phenotype (Supplemental Figure 4, A and B). These cells are less than 1% of total CD8^+^ T cells at 3 months after transplant for all patients; they increase to 2.4% 1 week after viremia in CMV PCR^+^ and remain somewhat elevated 12 months after transplant compared with PCR^–^ controls (CMV PCR^–^ = 0.2%, CMV PCR^+^ = 1.0%). These cells had increased expression of genes associated with exhaustion (*CTLA4*, *TIGIT*, *IL-10*, and *CD38*) but also activation (*MKI67*, *CCL3*, *TNFRSF9*, and *TYMS*).

### Platelet activation and concurrent IL-15 with CD56^bright^ NK cells identify previremic patients.

To identify immunological correlates that discriminate CMV PCR^–^ and PCR^+^ patients prior to and after CMV viremia, we used partial least squares (PLS) regression to generate a linear combination of predictors from the input variables to determine the effect size and direction for each variable predicting outcome (23). NK and T cell immunophenotypes were integrated with plasma analyte profiling and whole-blood transcriptomics. Modules of coexpressed genes were defined by weighted gene coexpression network analysis (WGCNA) and evidence of pathway enrichment per module was determined using Reactome (Table 2); further details of key genes per module are provided in Supplemental Table 1.

**Figure 5:**
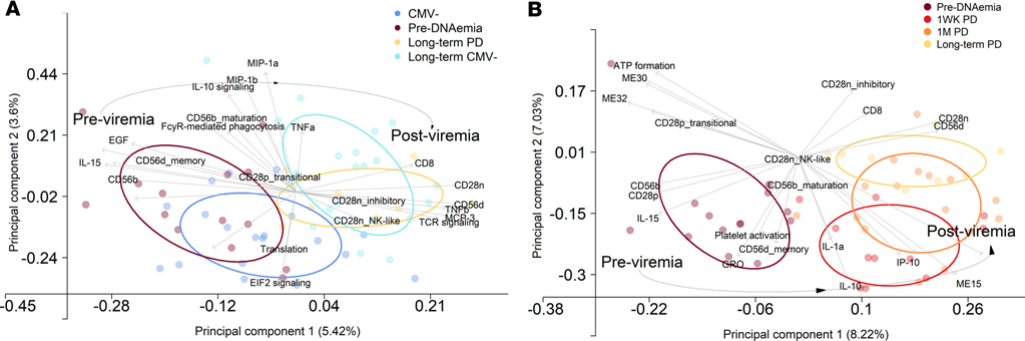
Partial least squares regression defines CMV PCR^+^ patients before and long-term after viremia and highlights acute and longitudinal immunological changes after detection of CMV viremia. Partial least squares (PLS) regression of NK and T cell phenotypes, plasma analytes, and whole-blood transcriptome modules was used to identify (**A**) variables important in differentiating CMV PCR^–^ and PCR^+^ patients longitudinally after transplant and (**B**) those important in defining immunological phenotypes prior to and longitudinally after detection of CMV viremia. Top 10 most informative variables per component are indicated by arrows and linked text. CMV PCR^–^ 3 months (dark blue, *n* = 17) and 12 months after transplant (light blue, *n* = 24), and PCR^+^ 3 months after transplant, before viremia, (purple, *n* = 14), 1 week after detection of viremia (1WK PV, red, *n* = 19), 1 month after detection of viremia (1M PV, orange, *n* = 9), and 12 months after transplant after viremia (yellow, *n* = 22) samples are indicated; ellipses capture 50% of the variance per group. Bold arrow highlights longitudinal progression immune profiles of CMV PCR^+^ patients. CD56b_maturation, CD56d_memory, CD28p_transitional, CD28n_inhibitory, and CD28n_NK-like were determined from protein surface expression profiles on NK and CD8^+^ T cells, as detailed in Figures 2 and 3.

CMV PCR^–^ and PCR^+^ patients at 3 months after transplant, as well as CMV PCR^+^ patients 3 and 12 months after transplant, could be distinguished primarily on the first principal component (Figure 5A). Three months after transplant, before detection of viremia, CMV PCR^+^ patients were defined by concurrently high levels of IL-15, EGF, and soluble CD40L in plasma with transcriptomic evidence of platelet activation. Additionally, the frequencies of CD56^bright^ NK cells and CD28^+^CD8^+^ T cells were at their highest levels 3 months before viremia. CMV PCR^–^ patients had increased evidence of cellular metabolic activity 3 months after transplant, with upregulation of translation and oxidative phosphorylation shown on the second principal component. Twelve months after transplant, PCR^+^ patients had expanded CD8^+^ T cells, particularly CD28^–^ subsets, and CD56^dim^ NK cells, with high levels of MCP-3 (CCL7) and TNF-β in plasma. Interestingly, upregulation of TCR signaling 12 months after transplant was associated with increased expression of inhibitory and NK-like receptors on CD8^+^ T cells, suggesting that these are functionally active. CMV PCR^–^ patients 12 months after transplant had some expansion of CD8^+^ T cells, but this was of lower magnitude, particularly with regard to CD28^–^CD8^+^ T cells, with additional upregulation of plasma TNF-α.

### CMV viremia promotes acute inflammation and long-term upregulation of inhibitory receptors on expanded CD8^+^ T cells.

PLS regression was similarly used to define immunological signatures at different stages of viremia in CMV PCR^+^ patients (Figure 5B). Three months after transplant, before viremia, and time points after detection of CMV viremia were separated on the first principal component. Samples before viremia were marked by high levels of plasma IL-15 and abundant CD56^bright^ NK cells and CD28^+^CD8^+^ T cells, as mentioned above. Platelet activation was demonstrated to be important, in concert with high levels of plasma GRO (CXCL1) and increased evidence of memory CD56^dim^ NK cells. These latter variables were more closely related to changes seen 1 week after viremia, suggesting that they may occur later than the aforementioned changes in IL-15 and NK and T cell subsets. All stages after detection of viremia again showed expansion of CD56^dim^ NK cells and CD28^–^CD8^+^ T cells; however, temporal differences were indicated by the second principal component. Patients 1 week after viremia had increased cellular activity, defined by upregulation of genes involved in oxidative phosphorylation, and a combination of inflammatory (IL-1α and IP-10 [CXCL10]) and regulatory (IL-10) plasma analytes. Whereas 12 months after transplant, longer-term after viremia, was delineated by increased abundance of total CD8^+^ T cells alongside continued expansion of CD56^dim^ NK cells and CD28^–^CD8^+^ T cell subsets, with upregulation of aforementioned inhibitory receptors on these T cells.

### NK cell and CD8^+^ T cell phenotype, but not IFN-γ and IL-10 signaling, predict effective control of CMV viremia.

**Figure 6:**
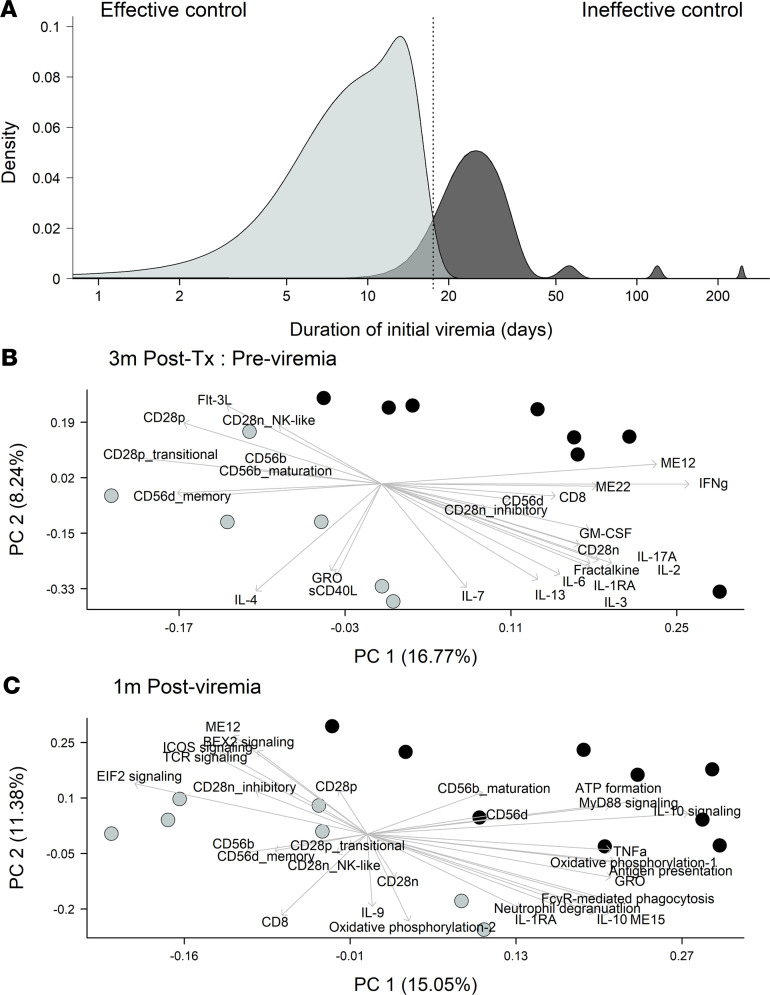
Cellular activity and NK and CD8^+^ T cell phenotype identify effective controllers of CMV viremia. (**A**) Duration of initial viremia in days was used to define patients as effective controllers (gray; duration ≤ 16 days, *n* = 12) and ineffective controllers (black; duration >16 days, *n* = 19). PLS regression of NK and T cell phenotypes, plasma analytes, and whole-blood transcriptome modules was used to identify variables predictive of control of CMV viremia (**B**) 3 months after transplant, before viremia, and (**C**) 1 month after detection of viremia. Top 10 most informative variables per component are indicated by arrows and linked text. Differential control of viremia is indicated by color of points. CD56b_maturation, CD56d_memory, CD28p_transitional, CD28n_inhibitory, and CD28n_NK-like were determined from protein surface expression profiles on NK and CD8^+^ T cells, as detailed in Figures 2 and 3.

CMV PCR^+^ patients had considerable heterogeneity in their CMV viremia. We used kmeans clustering to classify the ability of patients to control CMV viremia. Effective controllers (*n* = 12) resolved PCR-detectable viremia in ≤16 days and ineffective controllers (*n* = 19) failed to resolve viremia within 16 days (Figure 6A). PLS regression was employed as described above to identify immune profiles predictive of effective control of CMV viremia in PCR^+^ patients at 3 months after transplant, before viremia, and 1 month after detectable viremia. At both time points, there was significant variation in immune signatures associated with control of viremia. Composite immune profiles before viremia indicated that the ability to effectively control subsequent viremia was associated with upregulation of memory markers on CD56^dim^ NK cells (CD56d_memory), increased frequency of CD28^+^CD8^+^ T cells with a phenotype indicative of transition toward a CD28^–^ phenotype, and high levels of GRO and sCD40L in the plasma, previously associated with platelet activation (Figure 6B). Notably, high levels of IFN-γ in plasma before viremia was predictive of poor control of viremia. Cellular activity, specifically ATP formation and oxidative phosphorylation, was predictive of ineffective control 1 month after detectable viremia (Figure 6C). In support of this, EIF2 signaling, which regulates protein synthesis, was associated with effective control after viremia. Combined IL-10 and MyD88 signaling were associated with ineffective control, in combination with high levels of inflammatory plasma analytes such as TNF-α. In contrast, increased evidence of CD56^dim^ memory NK cells and expression of inhibitory and NK-like receptors on CD28^–^CD8^+^ T cells, and overall frequency of CD8^+^ T cells, were indicative of effective control of viremia.

## Discussion

This study utilized systems immunology, multiomic, and longitudinal profiling of kidney transplant recipients with and without evidence of CMV viremia to define immunological correlates of onset, progression, and control of viremia. Transplant recipients prior to detection of CMV by PCR could be identified by expansion of circulating immature CD56^bright^ NK cells, blood transcriptomic evidence of platelet activation, and high levels of plasma IL-15, EGF, and sCD40L. The acute stages of CMV viremia were characterized by downregulation of these factors, an imbalance of inflammatory and regulatory plasma analytes, and expansion of CD56^dim^ NK cells and CD28^–^CD8^+^ T cells. Continued expansion of these cells and upregulation of inhibitory and NK-like receptors on subpopulations of CD8^+^ T cells delineated time points further out from detection of viremia, with NK and CD8^+^ T cell phenotype also proving to be crucial in effective control of viremia in CMV PCR^+^ patients.

A novel finding of this study was the greater abundance of CD56^bright^ NK cells in CMV PCR^+^ patients at 3 months after transplant, before viremia, compared with CMV PCR^–^ matched controls, an immature subset previously described as having increased cytokine responsiveness but reduced killing potential (24). NK cells, a vital component of antiviral immunity, are known to be altered in terms of phenotype and function by CMV infection (6); however, these NK cells are of a more mature CD56^dim^ phenotype. Specifically, CMV-seropositive individuals have expanded memory-like NK cells expressing NKG2C, KIRs, and CD85j, with downregulation of natural cytotoxicity receptors (4, 25). These CD56^dim^ NK cells have enhanced killing activity, and their importance for control of CMV is further supported by the numerous methods that CMV has developed to evade NK cells (6). Increased abundance of immature CD56^bright^ NK cells before viremia was likely driven by high levels of plasma IL-15 observed before viremia, as IL-15 is known to preferentially expand CD56^bright^ NK cells (26–28). Expansion of this NK subpopulation has been described previously in CMV reactivation in acute leukemia patients following stem cell transplantation (29); however, this change was seen after detection of CMV in the periphery, whereas we observed an increase in CD56^bright^ NK cells prior to detection of viremia. Interestingly, in the context of acute leukemia, and other studies of cancer, increased abundance of these immature NK cells is beneficial in reducing relapse rates and improving survival (30, 31). Several studies have shown the importance of IL-12 and IL-18 in generating NK cell memory in response to CMV (32–34); however, these were not predictive of viral control in this study. Notably, the majority of the enrolled patients were CMV seropositive prior to transplant and, therefore, likely already had expanded NKG2C^+^ memory NK cells, which are less responsive to cytokine stimulation. This may explain the weak association between plasma levels of IL-12 and control of CMV viremia. In the context of kidney transplantation, the expansion before viremia of this immature NK subpopulation is unlikely to be beneficial due to the lack of a memory-like phenotype and the innate limited killing capacity of these cells. These potentially novel changes occurring before viremia instead suggest that IL-15–driven expansion of CD56^bright^ NK cells may be associated with impaired control of CMV primary infection and/or reactivation and may be an important early indicator of CMV viremia. Alternatively, this expansion of immature CD56^bright^ NK cells before viremia may provide an expanded population of the precursor cells for mature NK cells that subsequently work in conjunction with CD8^+^ T cells to resolve infection. This is supported by the observed longitudinal increase in number and relative abundance of mature, CD56^dim^ NK cells after viremia and their association with control of viremia. Whether these changes before viremia in NK cell phenotypes are beneficial or not, detection of them may present an earlier opportunity to preemptively treat high-risk recipients to prevent CMV viremia and disease.

Previremic patients additionally displayed upregulation of transcripts involved in platelet activation, concurrent with increased levels of plasma EGF and sCD40L. Studies in mice and in vitro human studies have shown that CMV infection of endothelial cells leads to increased platelet aggregation and recruitment of neutrophils (35), with direct activation of platelets induced by CMV through TLR2 (36). This direct activation in vitro led to secretion of proinflammatory sCD40L and IL-1b and proangiogenic VEGF by platelets, while work in mice additionally found increased extravasation of neutrophils linked to CMV-induced platelet activation (36). In contrast, there are reports of CMV infection being associated with low platelet counts (37, 38), potentially due to destruction of infected platelets or infection of platelet-producing megakaryocytes (37). In this study, transcriptomic and proinflammatory evidence of platelet activation may be indicators of ongoing viral replication, due to the direct nature of the interaction between CMV and platelets. This is supported by increased levels of EGF, as the EGF receptor is an important receptor for CMV (39, 40); its expression is modulated by multiple viral factors and is hypothesized to function in the switch between latency and reactivation (41). Temporally, these changes appeared to indicate a transitional stage between sampling before viremia and 1 week after viremia, further supporting platelet activity and associated proinflammatory factors as related to ongoing viral replication. This suggests a model in which IL-15–associated expansion of immature NK cells precedes peripheral CMV viremia, with subsequent activation of platelets indicating onset of viremia. Notably, proinflammatory indicators, including interferon signaling and plasma levels of IP-10, MIP-1α, and MIP-1β, were highest before and after viremia in patients who failed to control CMV and experienced long-duration and high-load infections. Therefore, in this study, a proinflammatory environment was indicative of ongoing CMV viremia but was not associated with effective control of the virus.

Long-term immunological dynamics after viremia were dominated by changes in number and phenotype of NK and CD8^+^ T cells. CD56^dim^ NK cells expanded after viremia, while long-term after transplant the ratio of CD56^dim^ to CD56^bright^ NK cells was equivalent between CMV PCR^+^ and PCR^–^ patients. Delayed repopulation of these memory-like CD56^dim^ NK cells has previously been associated with CMV disease in stem cell and kidney transplant recipients (13, 14, 42). There is also evidence of long-term persistence of these cells following viremia (43). This would support the previously described hypothesis that variations in NK cell subpopulations in the first few months after transplant, specifically, increased abundance of CD56^bright^ NK cells, are associated with differential susceptibility to CMV viremia. We also observed upregulation of memory-associated markers on CD56^dim^ NK cells in CMV PCR^+^ patients with viremia that resolved more quickly, further strengthening our hypothesis that NK cell function effects control of CMV.

CD8^+^ T cells, mostly CD28^–^, increased acutely and persisted long-term after viremia. An interesting finding of this study, in contrast to NK cells, was that PCR^+^ and PCR^–^ patients were not equivalent long-term in their CD8^+^ T cell profile. Even at 12 months after transplant, and several months after resolution of CMV viremia, CD8^+^ T cells were more abundant in PCR^+^ patients with an increased ratio of CD28^–^ to CD28^+^ cells. CD28^–^CD8^+^ T cells are known to increase with age and prior CMV exposure, with continued expansion over time, reportedly due to persistent antigen stimulation, termed memory inflation (9, 10, 44). CD28^–^CD8^+^ T cells are late-differentiated cells, known to express combinations of NK receptors, such as CD56, NKG2A, and KIRs, as well as inhibitory receptors and markers of terminal differentiation, including PD-1, CD57, and LAG3 (45). Expression of these inhibitory factors and increasing abundance with age have led to suggestions that these cells have reduced immunostimulatory capacity and are associated with immune senescence (46). While early studies found reduced proliferative ability compared with CD28^+^ cells (47), more recent studies have shown similar proliferative ability between CD28^+^ and CD28^–^CD8^+^ T cells (48, 49). Additionally, these cells have demonstrated cytotoxic ability and production of cytokines (47, 48). In this study, single-cell transcriptomics identified transcripts common to all CD28^–^CD8^+^ T cells, including *GZMB*, *NKG7*, and *ZEB2*, but also highlighted considerable heterogeneity within this population. For example, CD57 and CD160, which tracked similarly by flow cytometry, delineated 2 subpopulations of late-differentiated CD28^–^ cells. Differences in proliferative ability and broader functionality are likely to vary within these subpopulations, suggesting higher resolution phenotyping and functional analysis are required to fully understand these cells. In support of this, while we observed expansion of CD28^–^CD8^+^ T cells in CMV PCR^–^ patients, expression of NK and inhibitory receptors was not similarly upregulated, further suggesting different functional ability of these cells.

While CD8^+^ T cells are known to be important in controlling CMV and preventing associated disease in transplant recipients (50–53) and immunocompetent individuals (5), there have been inconsistent reports on the relationship between CD28^–^CD8^+^ T cells and kidney transplant outcomes (15, 16, 54, 55). This study highlighted a protective role for these terminally differentiated CD8^+^ T cells in resolution of CMV viremia where greater numbers of CD28^–^CD8^+^ T cells and expression of inhibitory and NK-like receptors on these cells were associated with effective control of viremia in CMV PCR^+^ patients. Expansion of these cells may be an indirect marker of viral control; alternatively, given the phenotypic similarity between them and described CMV-specific CD8^+^ T cells (7, 8), a subset may be directly killing CMV-infected cells. What is not known is how expansion of these cells, CMV-specific or not, affects broader host immunity. Circulating CD28^–^CD8^+^ T cells are expanded by other chronic viral infections, including EBV and HIV (56), but have also been implicated in poor responsiveness to influenza vaccination (57, 58) and promoting immunopathology in parasitic infections (59, 60). Additionally, tissue-infiltrating CD28^–^CD8^+^ T cells have been implicated in chronic hepatitis C–associated hepatocellular damage (61) and lupus nephritis (62). Long-term after viremia, an average of 65% of circulating CD8^+^ T cells of patients were CD28^–‑^. Previous studies of aging only reported levels this high in individuals over 80 years of age (63), suggesting that kidney transplant recipients who underwent CMV viremia in this study, ranging in age from 22 to 77 years, had significantly increased immunological age and possibly accelerated immunosenescence. If this proportion of circulating late-differentiated cells is maintained beyond 12 months after transplant, the endpoint of this study, they could have important implications for responses to natural infection and vaccination.

This study highlighted potentially new and important findings related to CMV viremia in kidney transplant recipients; however, we were not powered to identify differences between primary infection and reactivation due to limited cases of primary infection. It is plausible that observed increasing abundance of memory-like NK cells and CD28^–^CD8^+^ T cells after viremia, both known to expand long-term in latently infected individuals with CMV, may have differential dynamics in patients experiencing primary infection. A related limitation was the lack of pretransplant data on peripheral blood immunophenotypes. We found remarkable changes before viremia, approximately 3 months after transplant; however, it is possible that pretransplant heterogeneity in circulating NK and T cells may have affected the baseline parameters for patients in this cohort. Additionally, effective and ineffective control of CMV viremia was defined in this cohort using observed variance in duration of initial viremia; however, the majority of patients had low viral load and short duration of viremia by standard clinical definitions, suggesting most were controlling viremia well. We are prospectively enrolling a cohort of kidney transplant recipients enriched for CMV-seronegative individuals with CMV-seropositive donors in which we will validate the results of this study and explore in greater detail the differences between primary infection and reactivation. Finally, while we conducted high-resolution, multidimensional immunophenotyping of patients’ circulating cellular and soluble immune systems, we did not directly evaluate function of described NK and T cells. Single-cell transcriptomic profiling of ex vivo–isolated CD8^+^ T cells allowed us to analyze individual CD8^+^ T cells and provided invaluable insights into the heterogeneous profile of CD28^–^CD8^+^ T cells expanded after CMV viremia. We did not generate equivalent data for NK cells; therefore, there may be uncharacterized variability in CD56^bright^ and CD56^dim^ NK cells differentially affected by CMV viremia.

### Conclusions.

In this study, IL-15–associated expansion of immature CD56^bright^ NK cells was predictive of subsequent CMV viremia, while long-term expansion of memory-like CD56^dim^ NK cells correlated with effective control of viremia. This suggests that monitoring IL-15 levels and modifying NK cell function after transplant should be explored in the clinic as tools to detect and potentially limit CMV viremia. CD28^–^CD8^+^ T cells expanded and upregulated inhibitory and NK-associated receptors after viremia in patients who effectively prevented long-duration and high-load viremia. Single-cell transcriptomics highlighted remarkable heterogeneity in these late-differentiated CD8^+^ T cells, suggesting distinct subpopulations of CD28^–^ cells with variable function. These findings suggest signatures of innate activation may be prognostic for CMV reactivation or primary infection after transplant, while CD8^+^ T cell functionality, including NK-like phenotypes of CD28^–^CD8^+^ T cells, is critical for effective control of CMV and likely, therefore, limiting graft injury.

## Methods

### Study design and sample selection.

Transplant recipients enrolled in the University of California, Los Angeles, IRB-approved study from 2013 to 2015 were retrospectively reviewed for CMV viremia, and a subset of 62 patients were selected for study. This study included 31 kidney transplant recipients who had experienced CMV viremia (CMV PCR^+^); this was defined as CMV DNA >137 IU/ml in the patient’s blood by PCR and who had available plasma and PBMCs collected before detection of CMV PCR^+^ viremia (~3 months after transplant) and at approximately 1 week and 1 month after CMV^+^ PCR and 12 months after transplant (Figure 1). The commonly used term “viremia” is used herein to indicate the detection of CMV DNA in blood via PCR testing. Thirty-one kidney transplant recipients who had not experienced CMV viremia (CMV PCR^–^) were chosen as controls by nearest-neighbor matching based on propensity scores generated from a logit model using recipient age, sex, race, induction therapy, and CMV serostatus before transplant (Table 1). Control patients also had available plasma and PBMCs collected approximately 3 and 12 months after transplant. Whole blood was obtained at each visit. PBMCs and plasma were separated from whole blood upon collection and cryopreserved. All kidney transplant donors were CMV seropositive. Patient clinical care has been described previously (64). Patients received induction with either antithymocyte globulin (ATG) or basiliximab depending on pretransplant levels of sensitization and donor kidney quality. Patients were maintained on triple immunosuppression with tacrolimus and similar doses of mycophenolate mofetil and prednisone. CMV prevention was performed as follows: 6 months of valganciclovir for high-risk donor-positive (D^+^) and recipient-negative (R^–^) patients and 3 months of valganciclovir for intermediate-risk recipient-positive (R^+^) patients who received ATG induction. R^+^ patients who received basiliximab or low-risk (D^–^/R^–^) patients received acyclovir prophylaxis to prevent HSV and VZV infection. CMV PCR screening of peripheral blood for CMV DNA was performed every month while patients were on prophylaxis and at least annually following this, or if the patient developed symptoms concerning for CMV infection, such as fever, pancytopenia, or symptoms concerning for end-organ disease. CMV PCR testing was performed using the Cobas AmpliPrep/Cobas TaqMan CMV test (Roche) in the University of California, Los Angeles clinical laboratories.

### High-dimensional flow cytometric immunophenotyping of PBMCs.

For high parameter analysis using a multiparameter NK and T cell panel, 5 × 10^6^ PBMCs were stained in 96-well v-bottom plates using an optimized antibody panel, as described in Supplemental Table 2. Briefly, cells were washed with PBS, resuspended in 1 ml viability dye, and incubated at room temperature in the dark for 10 minutes. Cells were washed once with cold FACS buffer (PBS with 2% heat-inactivated FCS and 2 mM EDTA); resuspended in a staining cocktail that included the anti-γδ TCR antibody, human TrueStain FcX (BioLegend, 422302), and mouse serum (Jackson ImmunoResearch Labs, 015-000-001); and then incubated on ice for 10 minutes. Antibodies in the panel were sequentially added with 50 μl Horizon Brilliant Stain buffer (BD Bioscience, 56379) and then incubated on ice for 25 minutes. After washed with FACS buffer, samples were fixed in 100 μl Fluorofix Buffer (BioLegend, 422101). Samples were analyzed on a LSRFortessa X50 (FACSymphony) cytometer (BD Bioscience). Data were analyzed with FlowJo 10.7.1. NK and CD8^+^ T cells were gated as shown in Supplemental Figures 5 and 6, respectively.

### Single-cell RNA-Seq analysis of CD8^+^ T cells.

To obtain high-resolution transcriptomic profiling of CD8^+^ T cells, corresponding longitudinal PBMCs from 3 CMV PCR^+^ kidney transplant recipients and their matched CMV PCR^–^ controls were selected for further analysis from the 62-patient cohort described above. We chose CMV PCR^+^ patients with the greatest change in their proportion of CD8^+^ T cells expressing CD28 to maximize our ability to profile CD28^+^ and CD28^–^ cells. CD8^+^ T cells were negatively selected from thawed PBMCs using MACS magnetic beads. MicroBeads were similarly used for magnetic labeling and removal of cell debris, dead cells, and dying cells. Single-cell libraries of 4000 cells per patient were prepared using Chromium Single Cell 3’ kits (10X Genomics) before pooling and sequencing on the NovaSeq S2 instrument. Raw fastq files for gene expression (transcript) libraries were processed using Cell Ranger 5.0.1 to generate a gene expression count matrix. Data from all patients were integrated using the Cell Ranger function *aggr*. Data were quality controlled and analyzed in R, primarily using *Seurat*. Cells with >4000 or <200 unique features (genes) or mitochondrial counts >15% were removed. Cells identified as B, CD4^+^ T, or NK cells based on gene expression profile were removed prior to downstream analyses. Using the 3000 most variable genes and 50 principal components, 43 clusters of CD8^+^ T cells were identified. Genes upregulated or downregulated per cluster, compared with all other clusters, were identified using the *FindAllMarkers* function. Complete gene expression profiles of all cells were reduced to 2 dimensions using t-SNE analysis for visualization purposes with a perplexity value of 80.

### Plasma analyte profiling by 38-plex Luminex.

Plasma analyte profiling has been described previously (65). Briefly, we assayed plasma for cytokines and chemokines involved in stimulating neutrophils, antigen-presenting cells (i.e., CXCL1, CXCL2, CXCL5, CXCL8, CCL2, CCL5, CCL20, IL-1a, IL-1b, IL-17, IFN-γ, IFN I), T cells (GM-CSF, IFN-γ, IL-2, IL-4, IL-5, IL-13, and IL-17 among others), and B cells (INF-γ, IL-12, IL-2, IL-4, IL-13, IL-10, and TGF-β), using the 38-plex Luminex multibead arrays from Millipore. Raw MFI values were batch-corrected, using the ComBat algorithm, followed by log2-transformation to fit a normal distribution.

### Whole-blood transcriptomics by RNA-Seq.

RNA was isolated from 1 mL RBC-lysed whole-blood samples stored in RNAlater (Thermo Fisher Scientific). Globin RNA was removed using the GLOBINCLEAR kit (Thermo Fisher Scientific). Libraries were prepared for samples that passed quality control using the KAPA stranded-mRNA kit. RNA-Seq details and raw data processing details have been described previously (65). Briefly, prepared libraries were sequenced on the Illumina HiSeq3000 platform. After quality control by FastQC, reads were aligned to the GRCh38 human reference genome using STAR and count tables were generated using R package *Rsubread*. For this analysis, transcripts were included with a minimum count of ≥5 reads in ≥10% of samples. Modules of coexpressed transcripts were defined using WGCNA (66), with a minimum module size of 20 transcripts and merging of coexpression modules with correlation ≥0.75. Expression of each module was represented by the module eigengene, the first principal component of each module transcript expression matrix. Reactome was used to identify enriched pathways for each module.

### Availability of data and materials.

All sequencing data are available at GEO (NCBI) under accession GSE168598 (whole-blood RNA-Seq) and PRJNA745955 (CD8^+^ T cell single-cell RNA-Seq). Described 38-plex Luminex data, flow cytometry profiling of NK and T cells, and deidentified patient clinical and demographic information have been deposited in ImmPort (accession SDY1600).

### Statistics.

Univariate analysis of cell subsets and ratios with respect to clinical group was conducted by linear regression, including patient ID as a random effect variable. Models were considered significant if *P* values were less than or equal to 0.05. PLS regression (23) was used to compare multiple immune response variables to identify immune correlates of CMV viremia. PLS generated a linear combination of predictors from the input variables to determine the effect size and direction for each variable predicting outcome. Variables were integrated by PLS regression, with 10-fold cross-validation to determine the optimal number of components to retain. For visualization, the 10 most informative variables per component were included.

### Study approval.

We enrolled kidney transplant recipients after transplantation at Ronald Reagan Medical Center. The University of California, Los Angeles, Institutional Review Board approved this observational study (IRB#11-001387). All patients signed an informed consent.

## Author contributions

AH, MD, SB, JMS, DWG, MR, LLL, and EFR contributed to study design. HP, SS, JAH, KI, GS, ML, SB, JMS, MR, LLL, and EFR contributed to data collection. HP, SS, JAH, KI, YS, RP, RA, MR, LLL, and EFR contributed to data analysis. All authors interpreted the findings, contributed to writing the manuscript, and approved the final version for publication.

## Supplementary Material

Supplemental data

## Figures and Tables

**Table 1:**
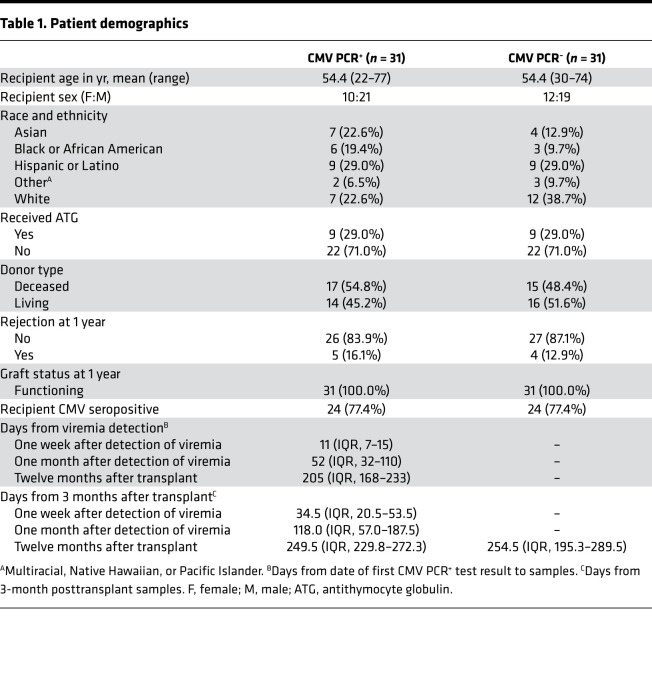
Patient demographics

**Table 2:**
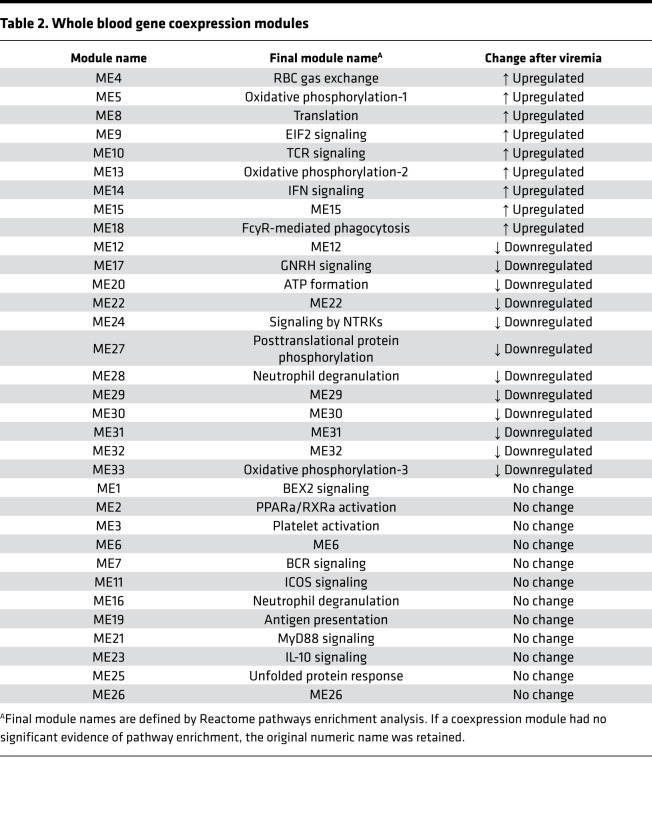
Whole blood gene coexpression modules

## Version 1 10/05/2021

In-Press Preview

## Version 2 11/08/2021

Electronic publication
