# Supplementary material for: NK and CD8+ T cell phenotypes predict onset and control of CMV viremia after kidney transplant
Source: JCI Insight. 2021 Nov 8;6(21):e153175. doi: 10.1172/jci.insight.153175 (PMC8663544; doi:10.1172/jci.insight.153175)
Supplement: Supplemental data [file jciinsight-6-153175-s196.pdf]

NK and CD8 T cell phenotypes predict onset and control of CMV viremia post-kidney transplant

Authors: Harry Pickering[1], Subha Sen[1], Janice Arakawa-Hoyt[2], Kenichi Ishiyama[2], Yumeng Sun[1], Rajesh Parmar[1], Richard S. Ahn[3,4], Gema Sunga[1], Megan Llamas[1], Alexander Hoffmann[4,5], Mario Deng[5], Mike Bunnapradist[5], Joanna M. Schaenman[5], David W. Gjertson[1,6], Maura Rossetti[1], Lewis L. Lanier[2+], Elaine F. Reed[1+*] and CMV Systems Immunobiology Group

Affiliations: [1]Pathology and Laboratory Medicine, University of California Los Angeles, Los Angeles, CA; [2]Department of Microbiology and Immunology, Parker Institute for Cancer Immunotherapy, University of California San Francisco, San Francisco, CA; [3]Microbiology, Immunology, and Molecular Genetics, University of California Los Angeles, Los Angeles, CA; [4]Institute for Quantitative and Computational Biosciences, University of California Los Angeles, Los Angeles, CA; [5]Medicine, University of California Los Angeles, Los Angeles, CA; [6]Biostatistics, University of California Los Angeles, Los Angeles, CA

*Corresponding author: Elaine F Reed, ereed@mednet.ucla.edu
+ L.L.L. and E.F.R. contributed equally to this work.

CMV Systems Immunobiology Group
Jenny Brook[1], Nakul Datta[2], Don J. Diamond[3], David Elashoff[1], Dmitry Rychkov[4], Minnie Sarwal[4], Tara Sigdel[4], Marina Sirota[4], Flavio Vincenti[4], Otto Yang[5]
[1]Department of Medicine Statistics Core, University of California Los Angeles; [2]Department of Surgery, University of California Los Angeles; [3]Department of Hematology & Hematopoietic Cell Transplantation, City of Hope; [4]School of Medicine, University of California San Francisco; [5]Department of Medicine, University of California Los Angeles

Supplementary Table 1 *Whole blood gene co-expression modules and key genes*

Module name	Total genes	ME representative genes[a]	Enriched pathway[b]	Pathway enriched genes[c]
ME1	162	ST6GAL1, ATM, LARS, MGA, PDS5A, IKZF1, ZNF609, CNTRL, PBRM1, MYCBP2	BEX2 Signaling Pathway	BAD,CDKN1A,MTOR,TSC1,VEGFA
ME2	186	TAOK1, RPS6KA3, DDX17, ERBIN, AVL9, TRAPPC8, RBL2, BICD2, BICRAL, PTPRC	PPARa/RXRa Activation	HSP90B1,IKBKB,MED12,NCOA6,PLCG2,PLCL2,SMAD2,SMAD4,TGFBR2
ME3	189	SPARC, ABLIM3, ITGA2B, CAVIN2, TUBB1, GNG11, ITGB3, PRKAR2B, ITGB5, PPBP	Platelet activation, signaling and aggregation	SELP,PROS1,MGLL,GP9,P2RY12,PF4,PPBP,MMRN1,EGF,SPARC,F13A1,MPIG6B,PDGFA,GNG11,CLU,PTK2,VCL,ENDOD1,VWF,CLEC1B,ABCC4,GP1BA,ITGA2B,ITGB3,RAB27B,TBXA2R
ME4	63	SLC25A39, BCL2L1, FBXO7, ALAS2, SNCA, DMTN, SELENBP1, EPB42, FAM210B, CA1	O2/CO2 exchange in erythrocytes	CA1,HBB,HBA2,SLC4A1
ME5	3154	TIAL1, PSMD2, VPS26C, TES, PIGS, PPP1R7, C1orf43, UBE2K, COPZ1, COX7A2L	Oxidative Phosphorylation	ATP5MC1,ATP5PF,ATP5PO,COX17,COX6A1,COX7A2,COX7B,COX8A,CYCS, NDUFA12,NDUFA2,NDUFA4,NDUFA5, NDUFA8,NDUFA9,NDUFB1,NDUFB3, NDUFB4,NDUFB5,NDUFS1,NDUFS2,NDUFS3,NDUFS4,NDUFS6,NDUFV2,SDHA,SDHD,SURF1,UQCR10,UQCRC2,UQCRFS1,UQCRH
ME6	2588	SF3B3, ELAC2, ESYT1, COPS7B, USP5, DNAJC11, RSAD1, METTL17, LRPPRC, ZNF740	NA	-
ME7	45	POU2AF1, IGHD, CD79A, IGHM, NIBAN3, CD19, CD22, BLK, PAX5, MS4A1	Signaling by the BCR	RASGRP3,BLK,BLNK,CD19,CD79B,CD22,CD79A
ME8	190	RPS6, RPS20, RPL30, RPS23, RPL35A, RPL31, RPS16, RPL3, RPL32, RPL10A	Formation of a pool of free 40S subunits	RPL22,RPL5,RPS27,RPS7,RPS27A,RPL31,RPL37A,RPL32,RPL15,RPL14,RPL24, RPL35A,RPL34,RPL37,RPS23,RPS14,RPS18,RPL10A,RPL30,EIF3H,RPS6,RPL35, RPS24,RPL41,RPLP0,RPS29,RPL36AL, RPS15A,RPL19,RPL38,RPL36,UBA52
ME9	72	RPL11, RPS15, NOP53, RPL13, RPS25, RPL29, RPL27A, RPL8, RPL18A, TLE5	EIF2 signaling	EIF3F,EIF3G,RPL11,RPL12,RPL13,RPL18A,RPL27,RPL27A,RPL28,RPL29,RPL8,RPLP1,RPS11,RPS15,RPS2,RPS25
ME10	98	PRKCH, ZAP70, PYHIN1, IKZF3, TSEN54, SBK1, FCRL6, CARD11, LCK, TBX21	TCR signaling	CARD11,CD247,CD3D,CD3E,CD3G,CD8A,LCK,NFATC2,ZAP70
ME11	327	BCL11B, LEF1, ITK, TRABD2A, IL7R, PLCG1, TCF7, PLEKHB1, NELL2, FAM102A	iCOS-iCOSL Signaling in T Helper Cells	CAMK4,CD28,CD40LG,ICOS,IL2RA,ITK,ITPR3,PIK3C2B,PLCG1,PLEKHA1,PRKCQ,TRAT1
ME12	40	MLLT6, TUBGCP6, TNRC6A, CCDC88C, ZBTB4, DNHD1,	NA	-

		CRTC3, PRRC2B, PCSK7, ANKRD52		
ME13	63	ATP5F1E, JTB, SF3B5, PSMB1, ATP5MG, PSMB4, SSR2, SNRPB, COX4I1, SERF2	Oxidative Phosphorylation	ATP5F1E,ATP5MG,ATP5PD,COX4I1,NDUFB6,NDUFB9,NDUFS5
ME14	136	IFIT3, UBE2L6, SERPING1, IFIT2, RSAD2, PARP9, TRIM22, EPSTI1, XAF1, STAT2	Interferon signaling	IFI35,IFI6,IFIT1,IFIT3,IFITM1,IFITM3,ISG15,MX1,OAS1,STAT1,STAT2,TAP1
ME15	18	VAMP5, FBXO6, PSMB8, PSTPIP2, TIFA, CALHM6, KPTN, TIMM10, FRMD3, CXorf21	NA	-
ME16	62	TCN1, CD24, MMP8, BPI, COL17A1, LTF, CEACAM8, RETN, LCN2, OLR1	Neutrophil degranulation	SLC2A5,LTF,PLAC8,ABCA13,CLEC5A,DEFA4,STOM,LCN2,TCN1,MS4A3,MMP8,OLR1,OLFM4,RNASE3,RNASE2,CTSG,ATP8B4,VAT1,MPO,SERPINB10,AZU1,PRTN3,ELANE,RETN,GPI,CEACAM6,CEACAM8,OSCAR,TRPM2
ME17	463	SPI1, EFHD2, GNAI2, FMNL1, TSC22D4, ARRB2, BRI3, PDLIM7, ZYX, TYROBP	GNRH Signaling	ADCY3,CALM1,EGR1,GNAI2,GRB2,MAP2K2,MAP3K11,PLCB2,PRKACA,PRKCD,PXN,RAC1
ME18	1433	RTN3, FBXL5, CAP1, NCF2, NADK, ARPC5, RPS6KA1, TALDO1, JAML, MSRB1	Fcγ Receptor-mediated Phagocytosis in Macrophages and Monocytes	ACTB,ACTG1,ACTR2,ACTR3,ARPC1A,ARPC3,ARPC5,CBL,CDC42,EZR,FCGR1A,FCGR2A,FCGR3A/FCGR3B,FGR,HCK,INPP5D,MAPK1,MAPK3,NCF1,PAK1,PIK3R1,PTEN,PTK2B,RAB11A,RAC2,SYK,TLN1,VAMP3,VAV1
ME19	1241	OSBPL2, STX3, ZDHHC18, NAMPT, MXD1, DENND5A, ACSL1, DENND3, FAM49B, UBE2B	Antigen presentation	KLHL21,UBR4,RNF220,FBXO11,CUL3,TRIP12,PSMD6,SEC61A1,TLR1,SEC31A,UBE2D3,TLR2,PJA2,x,MYLIP,RNF144B,HLA-G,HLA-A,HLA-E,HLA-C,HLA-B,CUL7,UBE2J1,FBXL13,UBE2H,MKRN1,LONRF1,LY96,HSPA5,ANAPC2,UBE2D1,ASB8,UBC,PSMC6,AREL1,BTBD6,STUB1,NPEPPS,RBCK1,ITCH
ME20	36	BRD4, ANKRD11, CREBBP, KMT2B, SPEN, SF3A1, CIC, EP300, SRRM2, ZMIZ1	Formation of ATP	ATP5MC3
ME21	504	B4GALT1, HIF1A, ETF1, MAPKAPK2, SYAP1, ELL, SAR1A, CHD1, KDM5B, AP1G1	MyD88 dependent cascade	MAPKAPK2,IRAK2,TNIP2,NFKB1,MEF2C,PPP2CA,PPP2CB,RIPK2,MAP3K8,TRAF6,RELA,MAPK7,NFKBIB,PPP2R1A
ME22	36	SRRM1, ELMSAN1, PRRC2C, WBP11, USP42, CBLL1, SUPT5H, ZBTB2, NOL4L, RREB1	NA	-
ME23	48	ICAM1, NFKBID, ZC3H12A, NFKBIA, NLRP3, NFKBIZ, MARCKS, PLAGL2, IER3, TICAM1	IL-10 signaling	IL1B,TNF,ICAM1
ME24	34	BMF, KLF10, ID2, TMEM189, SRC, DOT1L, GPR35, IL10RA, CYC1, MFSD2A	Signaling by NTRKs	SRC,ID2,ID1

ME25	14	SNHG15, SDC4, TRIP10, CLIC4, CLCF1, XBP1, SPHK1, PTGER2, NOCT, EZH2	Unfolded protein response	XBP1
ME26	36	FKBP15, CYBB, NCKAP1L, HDLBP, COLGALT1, BTK, ANXA4, HK1, AHCYL1, TM9SF4	NA	-
ME27	328	ARHGEF10L, PLXNB2, LRP1, ASGR1, TNS3, RASSF4, CD163, ANXA2, CD86, SCIMP	Post-translational protein phosphorylation	FAM20C,IGFBP7,GOLM1,LAMB2,FUCA2,VCAN,RCN1,GAS6,APOL3,NID1,CST3
ME28	83	CTSZ, PSAP, GRN, APLP2, TNFSF13, CD33, CFP, EMILIN2, CUX1, NKIRAS2	Neutrophil degranulation	CREG1,RAP2B,CD14,GM2A,PSAP,CD44,LYZ,LGALS3,NPC2,PKM,CTSH,GRN,GAA,CD33,CTSZ,ITGB2,CFP
ME29	50	ZC3H7B, PIEZO1, MEN1, MYBBP1A, GIT1, SYMPK, DNMT1, FYN, CRTC1, ENTPD6	NA	-
ME30	40	PIP5K1C, SMARCC2, TBC1D17, PTPN23, PRR12, TSC2, IFFO1, CC2D1A, SAFB2, AP3D1	NA	-
ME31	84	TCEA2, DPP7, HMG20B, EMC10, AKT1S1, FKBP2, TNFSF12, PTMAP4, C8orf82, HDAC10	NA	-
ME32	73	SF3A2, FBRSL1, ZNF414, MTA1, ZNF580, ATXN2L, TMEM160, IDUA, MAP3K10, ATP5F1D	NA	-
ME33	12	MT-ND4, MT-CO2, MT-ND1, MT-ND2, MTATP6P1, MT-CYB, MT-ATP8, MT-ATP6, MT-CO1, MT-CO3	Oxidative Phosphorylation	MT-ATP6,MT-CO1,MT-CO2,MT-CYB,MT-ND1,MT-ND2,MT-ND4

a; genes with expression pattern best representative of combined module of co-expressed genes, b; enrichment pathway defined by Reactome, c; genes identified in enriched pathways

Supplementary Table 2 *High dimensional flowcytometric panel*

Target antigen	Fluorescence	Source	Clone
CD3	APC-H7	BD Biosciences	SK7
CD8	BUV395	BD Biosciences	RPA-T8
CD14	BUV496	BD Biosciences	M5E2
CD16	BUV805	BD Biosciences	B73.1
CD19	BUV496	BD Biosciences	SJ25C1
CD27	BB660	BD Biosciences	M-T271
CD28	BB700	BD Biosciences	L293
CD45	AF700	Biolegend	30-F11
CD56	PE-CF594	BD Biosciences	B159
CD57	BUV615-P	BD Biosciences	PK136
CD85j	PE-Cy5	BD Biosciences	GHI/75
CD127	BV750	BD Biosciences	HIL-7R-M21
CD137	BV650	BD Biosciences	4B4-1
NKG2A	PE-Vio770	Miltenyi Biotec	REA110
NKG2C	PE	Biolegend	S19005E
CD160	FITC	BD Biosciences	BY55
CD161	BB790	BD Biosciences	DX12
PD-1	BUV737	BD Biosciences	EH12.1
CD294	BV480	BD Biosciences	BM16
NKG2D	BV711	BD Biosciences	1D11
NKp44	BV786	BD Biosciences	p44-8
NKp30	BUV563	BD Biosciences	P30-15
Gd-TCR	BV421	BD Biosciences	11F2
TCR Vd2	BV605	BD Biosciences	AB_2741719
CD158(KIR2DL1/DS1,3,5)*	APC	Miltenyi Biotec	11PB6
CD158(KIR2DL3)*	APC	Biolegend	DX27
CD158e(KIR3DL1)*	APC	BD Biosciences	DX9
Live cell dead	BV570	BD Biosciences	AB_2869635

*Anti-KIR antibodies that are conjugated with the same fluorescence were used as mixture.

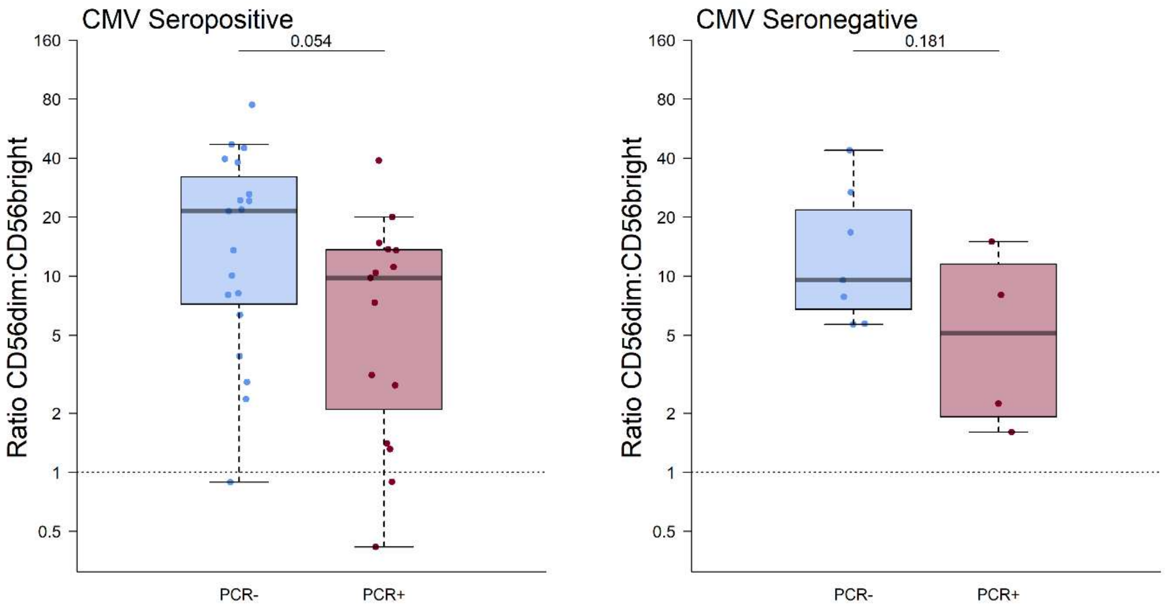

Supplementary Figure 1 *Ratio of CD56^{dim} : CD56^{bright} NK cells is consistently higher in CMV PCR- recipients at 3-months post-transplant, irrespective of CMV serostatus.* Ratio of CD56^{dim} : CD56^{bright} NK cells in CMV PCR- patients (dark blue, n = 17) and CMV PCR+ patients three-month post-transplant (purple, n = 14) in seropositive and seronegative recipients. P-values comparing CMV PCR- and CMV PCR+ at three-months post-transplant, determined by binomial logistic regression, are shown.

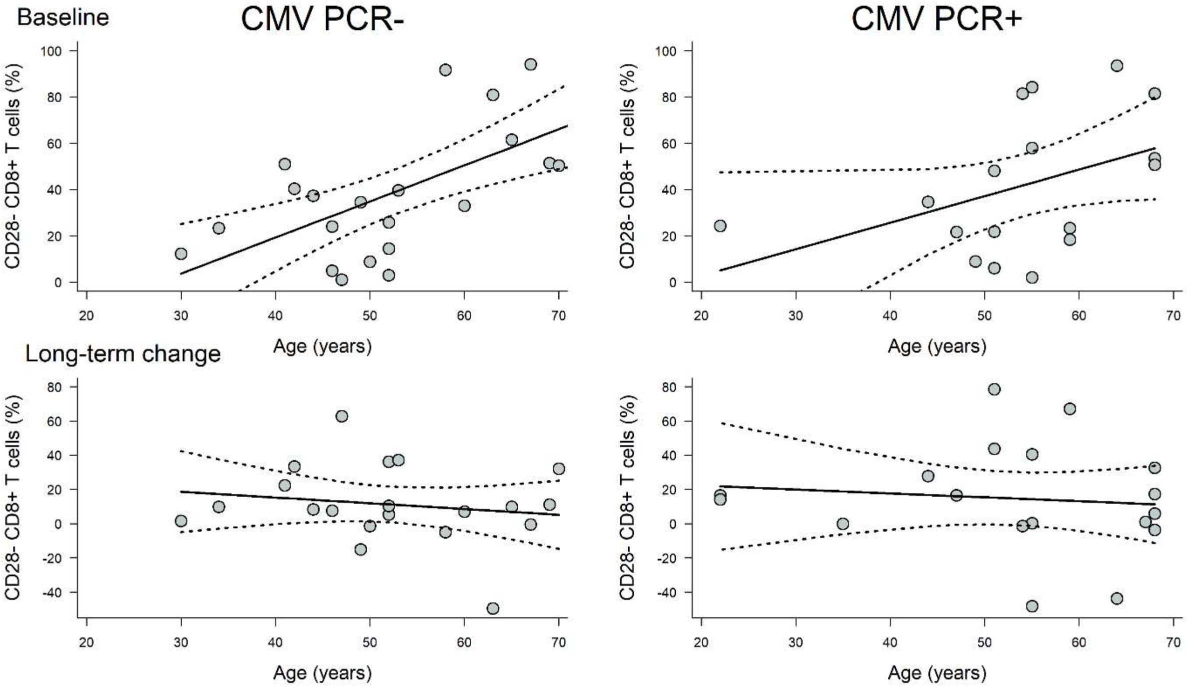

Supplementary Figure 2 *Long-term changes in CD28 expression on CD8 T cells are not age driven*. Baseline percentage of CD28⁻ CD8 T cells by age (years) in CMV PCR+ and PCR- patients. A linear regression of age on percentage of CD28⁻ CD8 T cells (line) and two standard deviations (dashed line) are shown. Long-term change in percentage of CD28⁻ CD8 T cells by age (years) in CMV PCR+ and PCR- patients, with serostatus similarly indicated. Long-term change was defined by subtracting percentage of CD28⁻ CD8 T cells at 3-months post-transplant from the percentage at 12-months post-transplant for each individual. Linear regression is shown as described above.

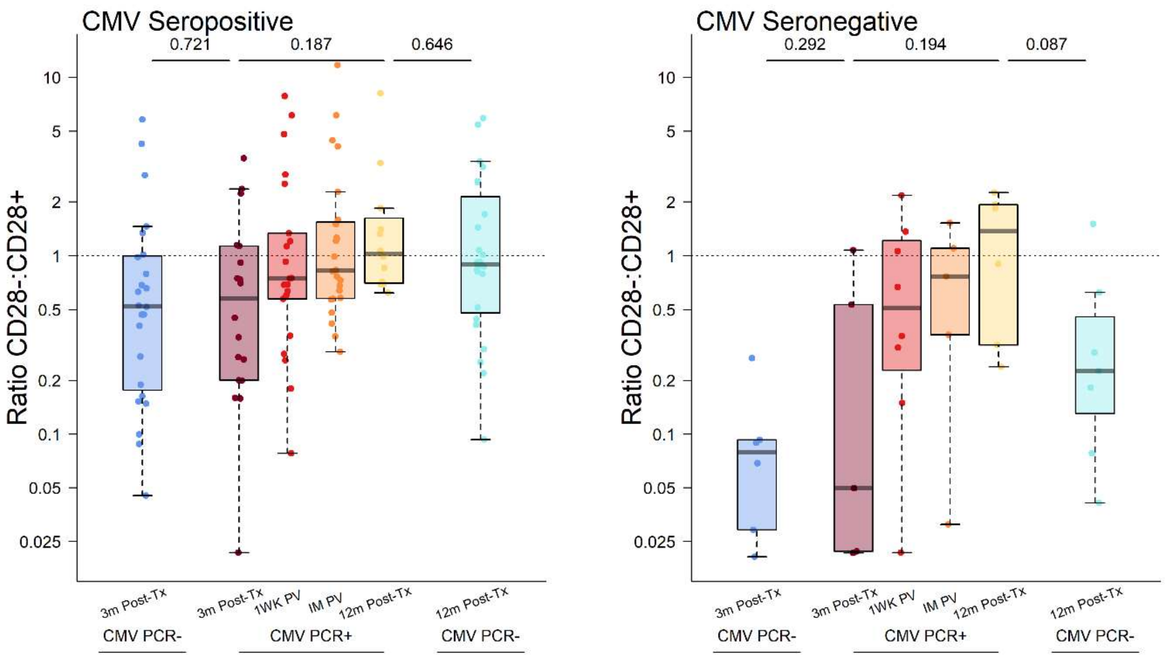

Supplementary Figure 3 *Ratio of CD28⁻ : CD28⁺ CD8 T cells increases post-viremia, irrespective of CMV serostatu*s. Ratio of $CD28^-$: $CD28^+$ CD8 T cells in CMV PCR- patients three-months (dark blue, n = 14) and twelve-months post-transplant (light blue, n = 24) and CMV PCR+ patients three-month post-transplant (purple, n = 19), one-week post-viremia (1WK PV, red, n = 9), one-month post-Viremia (1M PV, orange, n = 9) and twelve-months post-transplant (yellow, n = 22), in seropositive and seronegative recipients. P-values comparing CMV PCR- and CMV PCR+ at three- and twelve-months post-transplant, determined by binomial logistic regression, and change over time post-detection of viremia in CMV PCR+ patients, determined by linear regression including patient ID as a random effect, are shown.

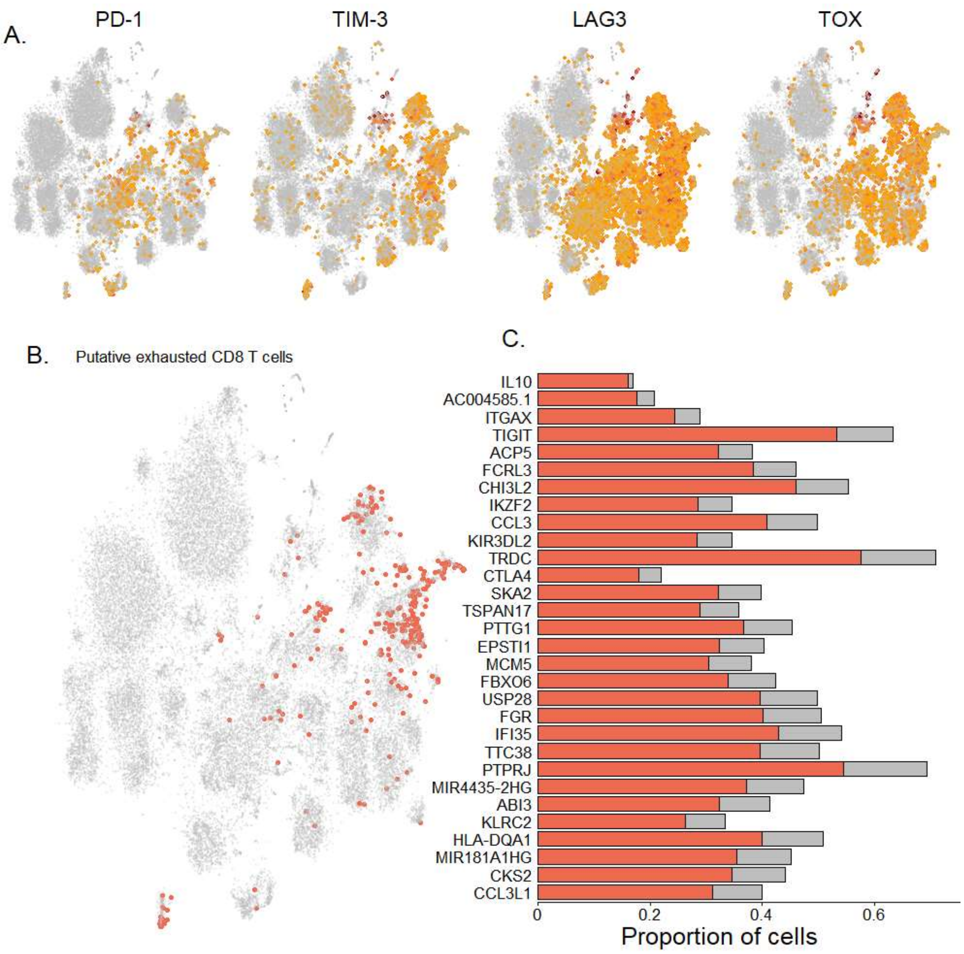

Supplementary Figure 4 *Putatively exhausted CD8 T cells*. A) t-SNE visualization of normalized expression of exhaustion-associated transcripts in single-cell transcriptomes of CD8 T cells, from low (grey) to high (red). B) Putatively exhausted cells (red), defined by co-expression of three or all four of PD-1 (*PDCD1*), TIM-3 (*HAVCR2*), *LAG3*, and *TOX*. C) Upregulated transcripts in putatively exhausted cells. Proportion of putatively exhausted cells expressing each transcript are shown (red) and proportion all other cells expressing each transcript (grey) are shown.

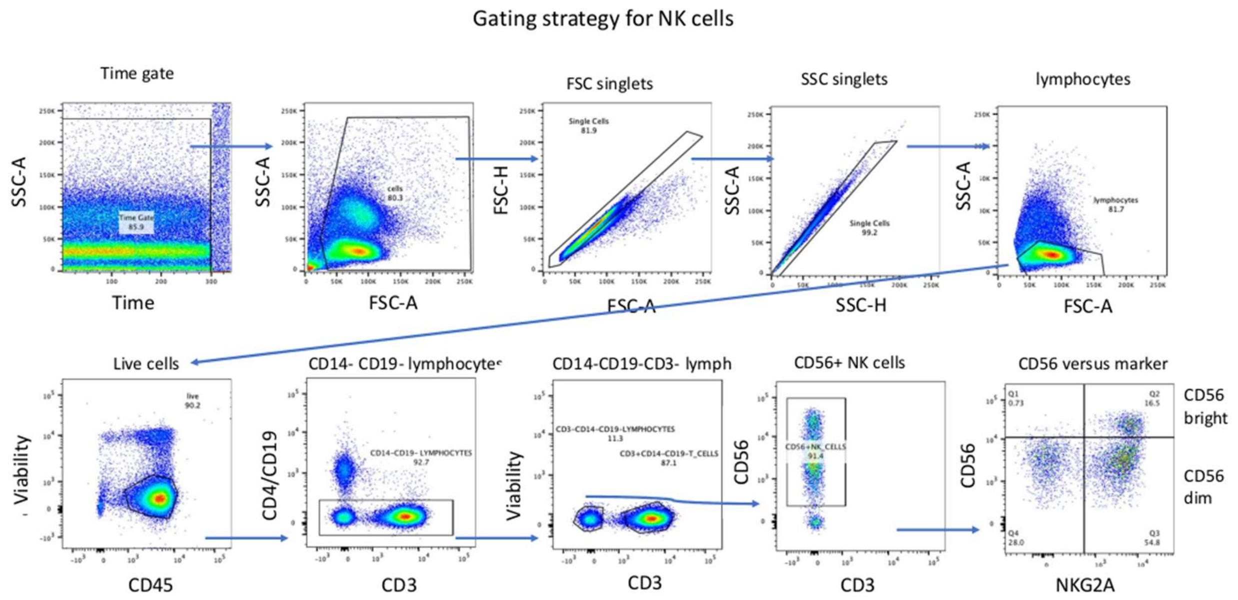

Supplementary Figure 5 *Example gating of CD56^{bright} and CD56^{dim} NK cells*.

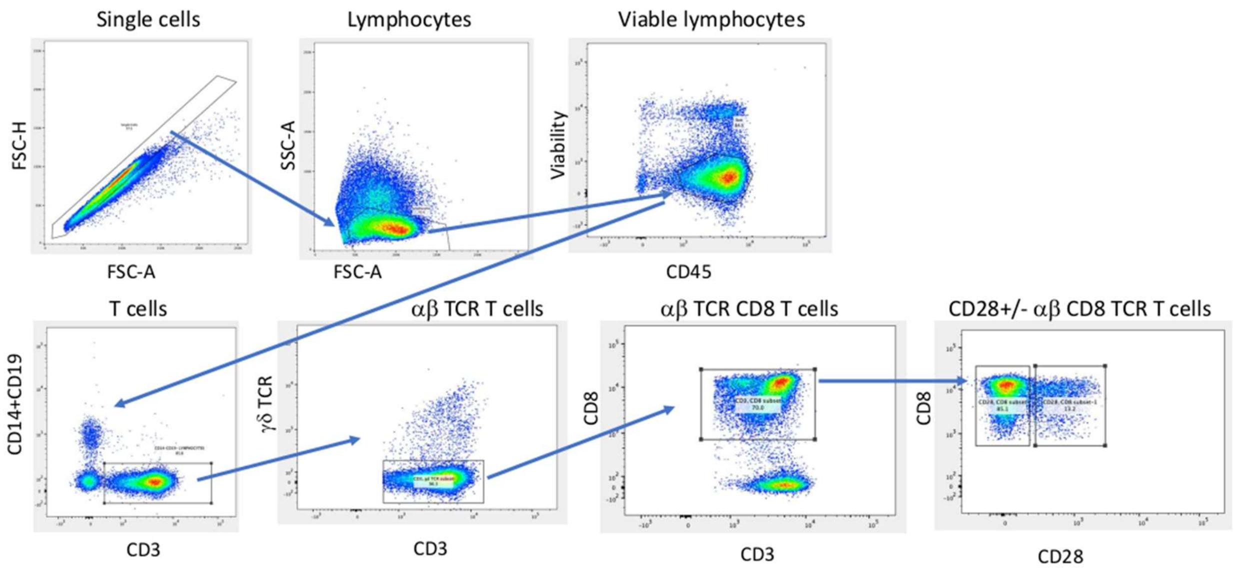

Supplementary Figure 6 *Example gating of CD28^{+} and CD28^{-} CD8 T cells*.
